# A Ligand Channel through the G Protein Coupled Receptor Opsin

**DOI:** 10.1371/journal.pone.0004382

**Published:** 2009-02-05

**Authors:** Peter W. Hildebrand, Patrick Scheerer, Jung Hee Park, Hui-Woog Choe, Ronny Piechnick, Oliver P. Ernst, Klaus Peter Hofmann, Martin Heck

**Affiliations:** 1 Institut für Medizinische Physik und Biophysik, Charité - Universitätsmedizin Berlin, Berlin, Germany; 2 Department of Chemistry College of Natural Science, Chonbuk National University, Chonju, South Korea; The Rockefeller University, United States of America

## Abstract

The G protein coupled receptor rhodopsin contains a pocket within its seven-transmembrane helix (TM) structure, which bears the inactivating 11-*cis*-retinal bound by a protonated Schiff-base to Lys296 in TM7. Light-induced 11-*cis*-*/all-trans*-isomerization leads to the Schiff-base deprotonated active Meta II intermediate. With Meta II decay, the Schiff-base bond is hydrolyzed, all-*trans*-retinal is released from the pocket, and the apoprotein opsin reloaded with new 11-*cis*-retinal. The crystal structure of opsin in its active Ops* conformation provides the basis for computational modeling of retinal release and uptake. The ligand-free 7TM bundle of opsin opens into the hydrophobic membrane layer through openings A (between TM1 and 7), and B (between TM5 and 6), respectively. Using skeleton search and molecular docking, we find a continuous channel through the protein that connects these two openings and comprises in its central part the retinal binding pocket. The channel traverses the receptor over a distance of *ca.* 70 Å and is between 11.6 and 3.2 Å wide. Both openings are lined with aromatic residues, while the central part is highly polar. Four constrictions within the channel are so narrow that they must stretch to allow passage of the retinal β-ionone-ring. Constrictions are at openings A and B, respectively, and at Trp265 and Lys296 within the retinal pocket. The lysine enforces a 90° elbow-like kink in the channel which limits retinal passage. With a favorable Lys side chain conformation, 11-*cis*-retinal can take the turn, whereas passage of the all-*trans* isomer would require more global conformational changes. We discuss possible scenarios for the uptake of 11-*cis*- and release of all-*trans*-retinal. If the uptake gate of 11-*cis*-retinal is assigned to opening B, all-*trans* is likely to leave through the same gate. The unidirectional passage proposed previously requires uptake of 11-*cis*-retinal through A and release of photolyzed all-*trans*-retinal through B.

## Introduction

The G protein coupled receptor (GPCR) rhodopsin contains 11-*cis*-retinal tightly bound in a binding pocket within the 7 transmembrane helix (7TM) bundle. In the dark, the retinal ligand acts as a strong inverse agonist and is covalently bound by a protonated Schiff-base to Lys296 in the last TM helix of the opsin apoprotein [1]. By absorption of a photon, 11-*cis*-retinal is isomerized to the all*-trans* configuration, resulting in the deprotonation of the retinylidene Schiff-base and the formation of the active, G protein-binding metarhodopsin II (Meta II) state. With Meta II decay and hydrolysis of the retinylidene Schiff-base, all-*trans*-retinal is released from its binding pocket [2]. Fresh 11-*cis*-retinal is provided through a complex metabolic cycle and is eventually delivered to the photoreceptor disc membrane. From the membrane, the hydrophobic ligand is selectively taken up by the opsin apoprotein to regenerate the light-sensitive 11-*cis*-retinal bound rhodopsin ground state [3]. Previous experimental work has suggested that uptake and release of retinal may proceed through different gates in the receptor structure [4], [5]. However available crystal structures of inactive rhodopsin states did not show obvious structural features of a gate through which the ligand could pass [1], [6], [7], [8]. Molecular dynamics simulations using the inactive structure resulted in several possible pathways for the egress of retinal [9]. The present study takes advantage of the recently solved structures of the ligand-free apoprotein opsin (Ops*) and the ligand-free opsin structure stabilized by a high affinity peptide derived from the C terminus of the α-subunit of the G protein (Ops*-GαCT). In these structures, the receptor is found in an active conformation, with the hallmark of an outward tilt of TM6 and structural rearrangements in conserved E(D)RY and NPxxY(x)_5,6_F regions [10], [11]. Both structures have in common that the 7TM bundle of opsin opens into the hydrophobic membrane layer through two holes which we term opening A (between TM1 and 7), and B (between TM5 and 6), respectively. We describe here a continuous channel through the protein that connects these two openings and investigate properties of the channel with respect to the passage of retinal. We will also discuss possible scenarios for the uptake of 11-*cis*- and release of all-*trans*-retinal.

## Results

### A channel through opsin

**Figure 1:**
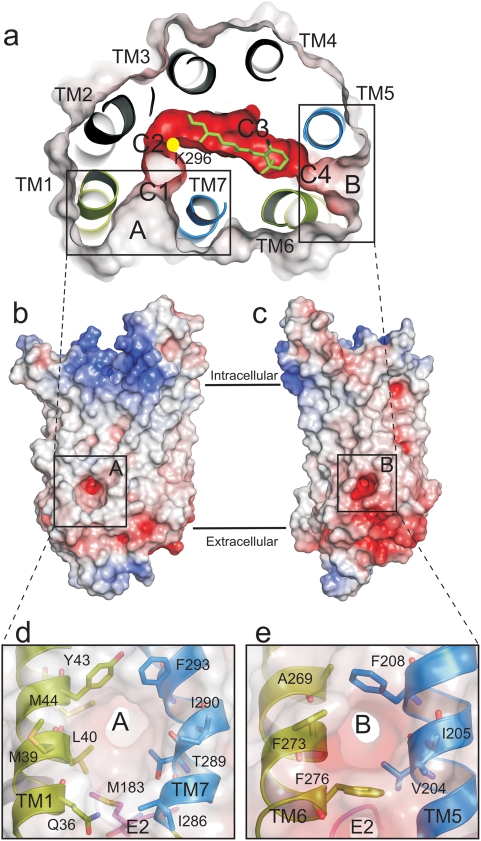
Structural features of the opsin ligand channel. Coplanar cut through opsin revealing the channel with opening A, B and constrictions C1-C4 (a, top view). The position of Lys296 is indicated by a yellow dot. All-*trans*-retinal (green) is docked into the binding pocket (see Methods). Electrostatic surface potentials were calculated using the program APBS [22] with nonlinear Poisson-Boltzmann equation and contoured at ±20kT/e and negatively and positively charged surface areas in red and blue, respectively (at high kT/e values the contour level is shifted from coloured to grey scale). (b, c) Side-views, with electrostatic surface potentials contoured at ±8kT/e. (d, e) Close-ups of openings A and B, defined by the residues as indicated.

The analysis starts from two crystal structures of opsin in its active conformation, namely the ligand-free apoprotein opsin, (Ops*, PDB entry 3CAP) [10] and opsin with the bound high affinity peptide (Ops*-GαCT, PDB entry 3DQB) [11]. Both structures display two well-defined openings between TM1 and 7 (opening A), and between TM5 and 6 (opening B), respectively. Using skeleton search algorithms [12] we find a channel through the protein that connects the retinal binding pocket with these two openings (Figure 1a). The channel contains characteristic constrictions which include a kink near the retinal binding residue, Lys296, where the conformation of the lysine side chain determines the channel clearance (see below).

**Figure 2 pone-0004382-g002:**
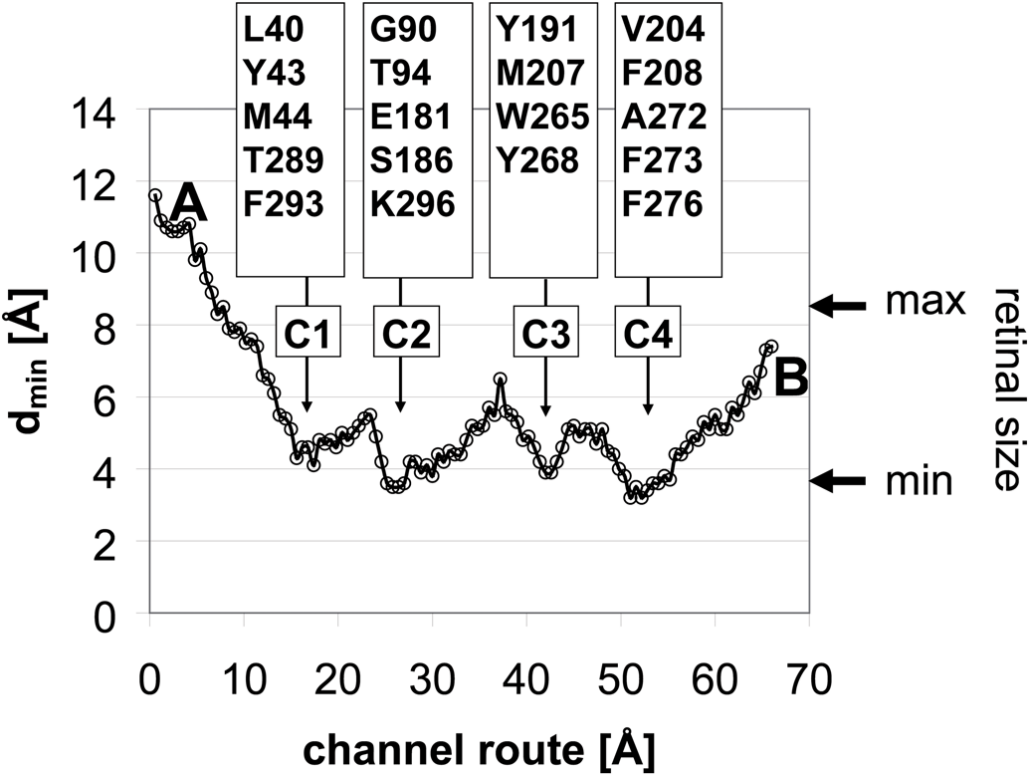
Constriction sites within the channel. Minimum inner width (d_min_) measured at intervals of 0.6 Å progressing from opening A to opening B. The residues defining the constriction sites (C1–C4) are indicated, as well as the maximum and minimum extensions of the β-ionone moiety of the retinal.

The channel traverses the receptor perpendicular to the membrane normal over a distance of 65-70 Å, coplanar with the retinal binding pocket. Both openings are funnel shaped (Figure 1a–c) and lined with aromatic residues (Figure 1d–e), namely Tyr43, Phe293 in the wider opening A and Phe208, Phe273 and Phe276 in opening B. In contrast to the hydrophobic openings the central part of the channel, which includes the retinal binding pocket, is clearly polar (Figure 1a). The minimum inner width of the channel varies from 11.6 Å at opening A to 3.2 Å at Lys296, its smallest constriction (Figure 2). A total of four constrictions (C1–C4) can be identified within the channel. These sites are so narrow that they have to be stretched to allow passage of the retinal β-ionone-ring. C1 and C4 are located at openings A and B, respectively, while C3 is located at Trp265 within the retinal binding pocket. A striking feature of the channel is C2, located at a 90° elbow-like kink, and close to the active site Lys296 (Figure 1a: C2). Notably, a polar cavity (Figure 1a, NC) is located between C2 and C1, which forms an appendix like extension towards the intradiscal side of the receptor. Along almost its full length, between C1 and C4, the floor of the channel is provided by the intradiscal loop E2, which is part of the “retinal plug” [13].

### Constriction of the channel at the active site

**Figure 3:**
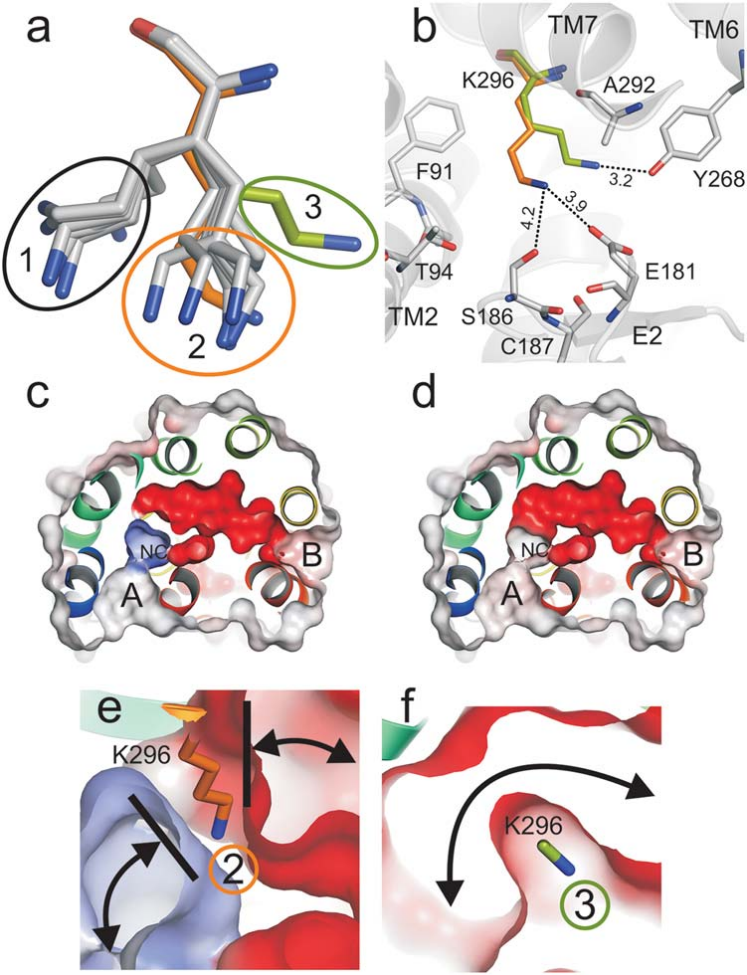
Effect of Lys296 conformation on channel constriction. (a) Clusters (1–3) of calculated Lys296 rotamers. Orange (cluster 2), calculated rotamers of Lys296 as also found in the crystal structure of Ops*-GαCT (PDB entry 3DQB). Light green (cluster 3), calculated conformation of Lys296 as used for skeleton search (see text for details). (b) Superposition of the two most plausible conformers of Lys296 shown with neighbouring residues (distance <5 Å) and with the potential network of hydrogen bonds (dashed lines). (c) View onto the ligand channel with the channel-closing conformation of Lys296 (cluster 2) hydrogen bonded to Ser186 and Glu181 and (d) in the channel-opening conformation hydrogen bonded to Tyr268 (cluster 3). Electrostatic surface potentials contoured at ±20kT/e, and negatively and positively charged surface areas in red and blue, respectively. Note that the positive charge of the ε-amino group of Lys296 (not shown) results in a positive surface potential of the neighbouring cavity (NC) in c, but is above the cut in d. Close-up view of Lys296 in (e) channel-closing (orange side chain – cluster 2) and (f) channel-opening conformation (light green side chain cut at the ε-amino group–cluster 3).

Lys296 is the only channel lining residue in the ligand-free Ops* structure for which no defined electron density was found, arguing for a high degree of flexibility [10]. To monitor the conformation of Lys296, 16 different rotamers have been generated [14], [15] and energetically minimized [16]. A population of six rotamers (Figure 3a, cluster 1) was excluded by the minimization process because they result in a clash of Lys296 side chain with Phe91. The remaining stable rotamers can be grouped in two populations: The first includes the conformation of Lys296 found in the crystal structure of Ops*-GαCT (Figure 3a, cluster 2, orange). This rotameric state allows a network of weak interactions between the ε-amino group of Lys296 and the side chains of Ser186 and Glu181 (Figure 3b). With this conformation the channel is blocked (Figure 3c, e). In another conformation (Figure 3a, cluster 3, light green) Lys296 is hydrogen bonded to Tyr268. Only in this case, C2 adopts an inner width of 3.8 Å and a continuous channel is formed (see Figures 1, 3d, f).

### Snapshots from a putative passage of retinoids

**Figure 4:**
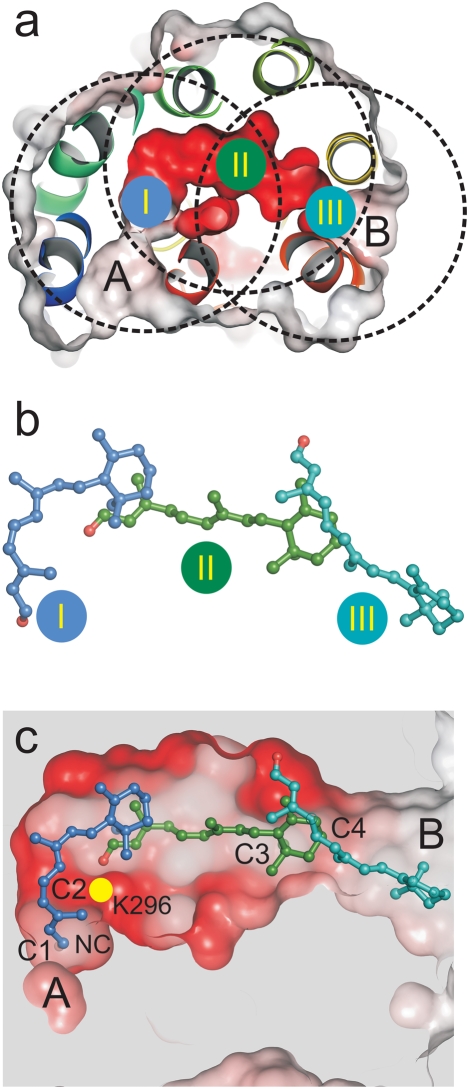
Location of the retinal docking sites. (a) The docking sites (I, II, III) are restricted to all residues within the radius of 10 Å (circles) from Met44 (I), Tyr268 (II) and Ala269 (III), respectively. Site I is close to opening A and the 90° kink of the channel, site II represents the retinal binding pocket, and site III is close to opening B. (b) Selected final conformations of retinal isomers resulting from the three docking procedures (see Figures 5–7 for details). Blue, 11-*cis-*retinal docked to site I; green, all-*trans*-retinal docked to site II; and cyan, all-*trans*-retinal docked to site III. (c) View onto the ligand channel (electrostatic surface potentials as in Figure 1a) with docked 11-*cis*- (blue) and all-*trans*-retinal (green, cyan). Parts of the receptor were omitted to visualize the two openings (A and B), the constrictions (C1–C4) and the neighbouring cavity (NC).

We performed docking experiments to explore low energy positions for 11-*cis*- and all-*trans*-retinal within the channel. To generate plausible snapshots along the putative ligand path, three docking sites were defined within the channel as described in Figure 4a, namely (i) docking site I spanning opening A and the 90° kink of the channel, (ii) site II, the retinal binding pocket and (iii) site III located close to opening B of the channel. Importantly, Lys296 is allowed to rotate freely in all the docking simulations carried out.

**Figure 5:**
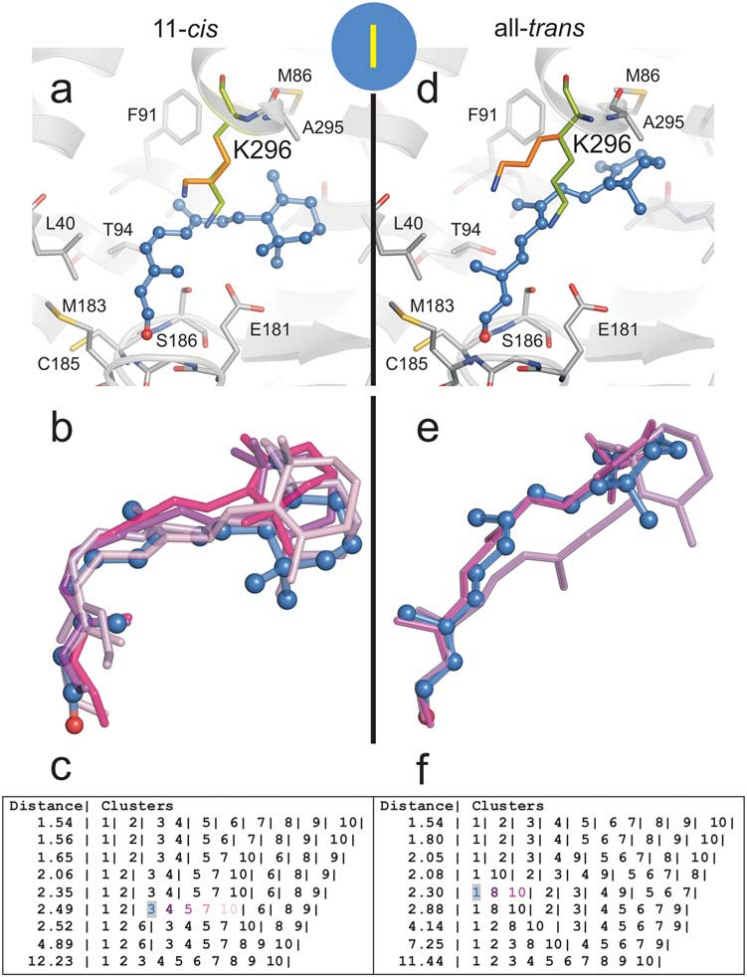
Docking of retinal isomers to docking site I. Flexible docking of (a–c) 11*-cis-*retinal and (d–f) all-*trans*-retinal to site I located between opening A and C2 at the 90° kink of the channel (see Figure 4). The crystal structure of Ops* (PDB entry 3CAP) was used and full flexibility for Lys296 side chain was allowed. The most likely conformation of (a) 11*-cis-*retinal and (d) all-*trans*-retinal is shown together with the neighbouring residues. The conformation of Lys296 obtained by the docking procedure (orange) is superimposed to the starting conformation (light green). Cluster of docking poses of (b) 11*-cis-*retinal and (e) all-*trans*-retinal and (c, f) the respective lists of ranked docking poses at different RMSD cut-off values (different colours identify individual poses). The best scored pose of the finally selected cluster (shaded) is shown with ball and sticks in a, b, d and e.

**Figure 6 pone-0004382-g006:**
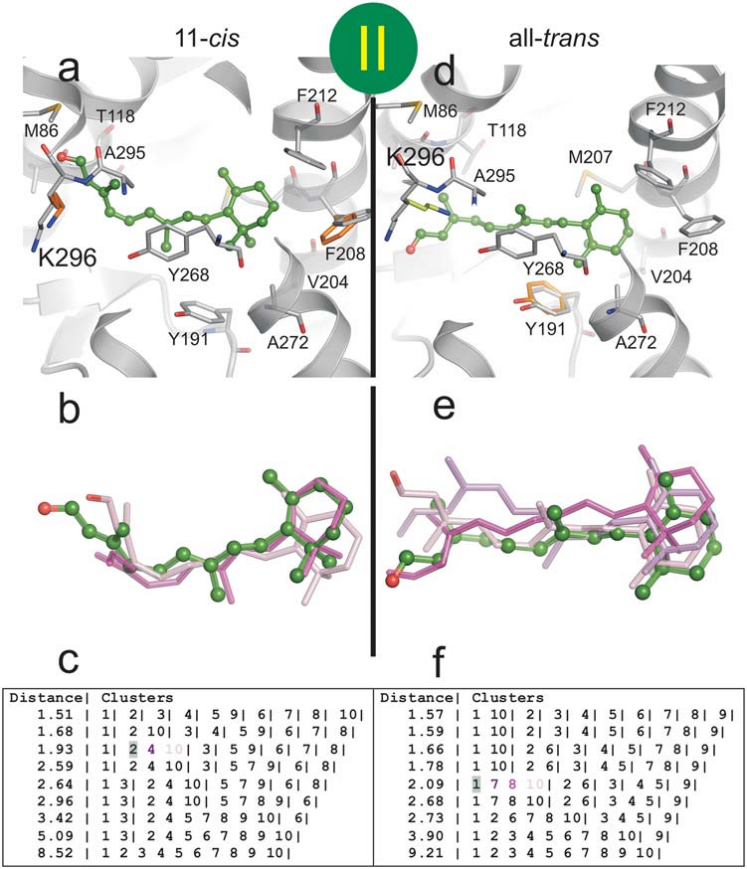
Docking of retinal isomers to docking site II. Flexible docking of (a–c) 11*-cis-*retinal and (d–f) all-*trans*-retinal to site II, i.e. the retinal binding pocket (see Figure 4). The crystal structure of Ops* (PDB entry 3CAP) was used and full flexibility for Tyr191, Val204, Phe208, Phe273 and Lys296 side chains was allowed. The most likely conformation of (a) 11*-cis-*retinal and (d) all-*trans*-retinal is shown together with the neighbouring residues. The residues with altered conformation (orange) are super­imposed to the starting conformation. Cluster of docking poses of (b) 11*-cis-*retinal and (e) all-*trans*-retinal and (c, f) the respective list of ranked docking poses at different RMSD cut-off values (different colours identify individual poses). The best scored pose of the finally selected cluster (shaded) is shown with ball and sticks in a, b, d and e.

**Figure 7:**
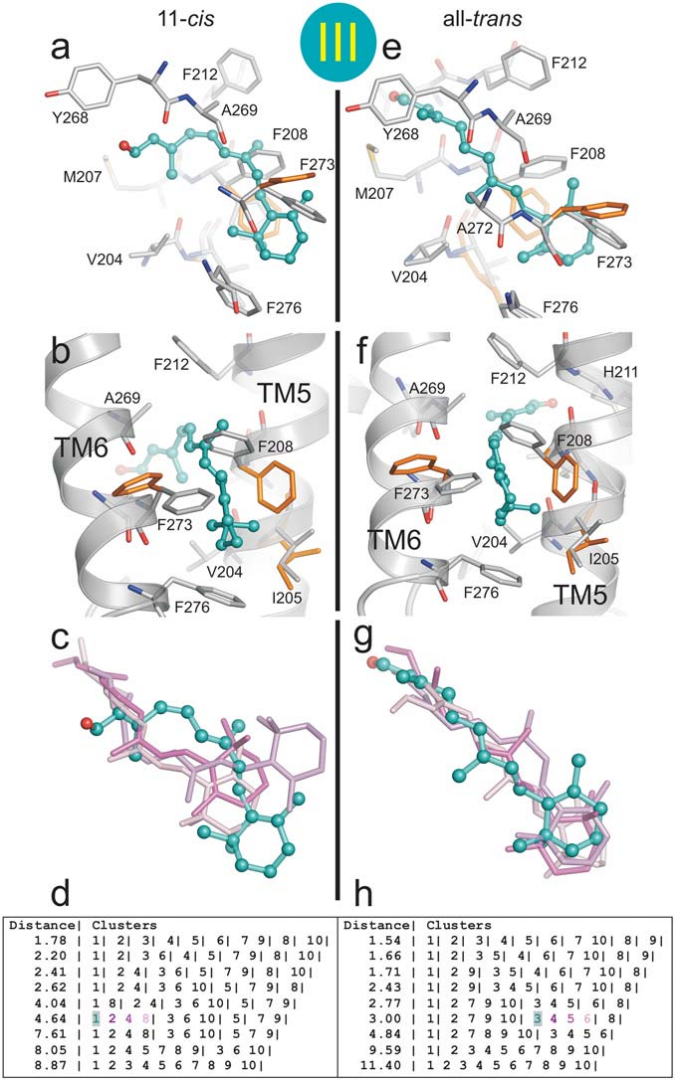
Docking of retinal isomers to docking site III. Flexible docking of (a–d) 11*-cis-*retinal and (e–h) all-*trans*-retinal to docking site III located close to opening B of the channel (see Figure 4). The crystal structure of Ops* (PDB entry 3CAP) was used and full flexibility for Tyr191, Ile205, Phe208 and Phe273 side chains was allowed. The most likely conformation of (a, b) 11*-cis-*retinal and (e, f) all-*trans*-retinal shown with neighbouring residues from two different perspectives (TM5 and TM6 are depicted in cartoon representation). The residues with altered conformation (orange) are superimposed to the starting conformation. Cluster of docking poses of (c) 11*-cis-*retinal and (g) all-*trans*-retinal and (d, h) the respective list of ranked docking poses at different RMSD cut-off values (different colours identify individual poses). The best scored pose of the finally selected cluster (shaded) is shown with ball and sticks in a–c and e–g.

In Figures 4b, c and 5–7 the clusters and the finally selected docking modes for 11-*cis*- and all-*trans*-retinal are shown at the three docking sites with the contacting residues of the receptor (distance cut-off: 5 Å). Docking of the two retinal isomers to docking site I reveals that the bent 11-*cis*-retinal nearly perfectly fits into the 90° kink of constriction site C2 (Figure 4c, Figure 5, left row). In this position the aldehyde moiety of the retinal is located in the polar cavity described above. All-*trans-*retinal can also be docked to docking site I (Figure 5, right row) but cannot adopt the kink due to its stretched conformation. Both retinal isomers fit well into docking site II with their native longitudinal orientation (i.e. with the aldehyde moiety orientated towards Lys296, Figure 6). This requires rendering the side chains of Tyr191, Val204, Phe208, Phe273 and Lys296 flexible. Both 11-*cis-* and all-*trans-*retinal can bind to docking site III and thus opening B when side chains of Tyr191, Ile205, Phe208 and Phe273 are rendered flexible (Figure 7).

## Discussion

### The channel as a property of the active conformation

Depending on the conformation of Lys296, a continuous channel is formed which connects the two openings found in the crystal structures of opsin (Figure 1–3). With its general architecture, the channel provides a basis for discussing the mechanism of uptake and release of the retinal 11-*cis* and all-*trans* isomers, respectively. The openings are found in the active conformation, in which opsin is competent to bind and activate the G protein transducin. Because the openings are absent in the inactively locked 11-*cis*-retinal bound dark state, we assume that the channel is a specific property of the active conformation. Hallmarks of Ops* include an outward tilt of TM6 and structural rearrangements in conserved E(D)RY and NPxxY(x)_5,6_F regions [10], [11]. Consistently, available functional data suggest that the uptake and release of retinal is facilitated by an active conformation, because 11-*cis*-retinal transiently stabilizes an active state of opsin in the course of regeneration [17], and the release of all-*trans*-retinal is favored with the pH dependence of the active Meta II state [18].

### Release of all-trans-retinal

A U-turn of retinal within the narrow channel seems very unlikely (Figure 2). Accordingly, the orientation of the retinal in its binding pocket, as known from the dark state rhodopsin structure[1], determines its longitudinal orientation during uptake and release. The low energy positions for 11-*cis*- and all-*trans*-retinal within the opsin channel obtained by molecular docking are snapshots that allow some conclusions about possible retinal paths. Passage of the stretched all-*trans*-retinal through the narrow 90° kink of the channel at Lys296 would require major conformational rearrangements of the overall opsin structure (Figure 5). We may thus conclude that photolysed all-*trans*-retinal is released through opening B and with the β-ionone-ring first. The docking experiments show that the binding pocket in the opsin structure can actually accommodate all-*trans*-retinal after hydrolysis of the covalent linkage to Lys296 (Figure 6). The β-ionone-ring of the retinal is then located between C3 and C4 (Figure 1a, 4c). The hydrophobic interaction between the ring and the aromatic side chains from opening B (Figure 1e) may guide the retinal into the lipid phase of the membrane [4]. Indeed, an intermediate state in which the β-ionone-ring of all-*trans*-retinal has passed C4 and interacts with the aromatic residues Phe208, Phe273 and Phe276 can be described by docking (Figure 4c, 7).

### Uptake of 11-cis-retinal

For the uptake of 11-*cis*-retinal, either routes through opening A or B can be envisaged. If we assume uptake through B, as discussed in Ref. [10], 11-*cis*-retinal must pass through the channel with the aldehyde moiety first. However, the hydrophobic nature of opening B and the likely orientation of the amphiphilic retinal within the membrane plane argue that the β-ionone-ring initially interacts with this site. Accordingly, the retinal has to flip upon uptake, which requires larger structural changes, including the breakage of a hydrogen bond between Gln279-Glu201 and a reorientation of the intradiscal loop E2 [10]. If we assume no U-turn is allowed and the all-*trans*-retinal cannot take the turn at the kink, this route would further imply that the release of the photolysed all-*trans*-retinal proceeds through the same opening.

Uptake of 11-*cis*-retinal through opening A again implies an orientation of the retinal with the β-ionone-ring passing first. The aromatic and hydrophobic residues around opening A (Figure 1d) would thereby assist the capture of the retinal during its uptake from the membrane and the retinal would already be in the right orientation. The transfer of 11-*cis*-retinal from opening A to the binding pocket then requires the complete passage of the polyene chain through the C2 kink (Figure 4c), before the retinylidene Schiff-base can form. The different docking modes for the two retinal isomers (Figure 5) indeed suggest that only the bent 11-*cis*-retinal can take the turn, but even this isomer needs the extra space provided by the neighboring cavity (Figure 4c, NC), and the flexibility of Lys296 (Figure 3). The longitudinal length of retinal was suggested earlier to account for the geometric specificity of opsin [19], [20]. In any case, the kink at Lys296 appears to be the main constriction site where 11-*cis*-retinal is selected against all-*trans*-retinal.

### Conclusion

The salient result of this study is that the available structural information can be used to identify a channel through the 7TM bundle of the opsin receptor. The conformational state of opsin's active site, Lys296, determines whether the channel is continuous or divided into two half channels. In terms of rhodopsin regeneration, it will now be essential to know how the two parts of the channel are linked and to obtain conclusive evidence whether the overall process is indeed unidirectional, i.e. whether both of the openings identified are functional gates through which retinal is taken up and released. Ways to elucidate the underlying mechanism include site directed mutagenesis combined with computational approaches. As to the generalization of these results to other GPCRs, rhodopsin may at first appear as a special case because it is provided with energy for retinal channeling by light-induced isomerization of the ligand. However, sources of energy for ligand channeling may also exist in other GPCRs, for example provided by phosphorylation and arrestin binding.

## Methods

### Receptor set-up

To generate snapshots along the putative ligand path, three binding sites were defined within the channel of the crystal structure of Ops* (PDB entry 3CAP, Figure 4a). These binding sites are defined as the receptor regions within the radius of 10 Å centred on Met44 (opening A, 90° kink of the channel), Tyr268 (retinal binding pocket) and Ala269 (opening B). Side chains of channel lining residues Lys296, Tyr191, Val204, Ile205, Phe208 and Phe273 were rendered flexible during the docking simulations (Figures 5–7). Water molecules and lipids were deleted in advance of the dockings. All hydrogen atoms, including those necessary to define the correct ionization and tautomeric states of residues such as Asp, Glu and His are added automatically by GOLD [21]. Thus, e.g. Lys296, the retinal attachment site, is protonated. No other manipulation was performed to the crystal structure.

### Ligand set-up

Structures of 11-*cis*- and all-*trans*-retinal, were downloaded from the freely available database of chemical compounds (http://pubchem.ncbi.nlm.nih.gov/) and energetically minimized with help of the force field GROMOS 43B1 to yield ligand structures close to their local potential-energy minima [16]. The starting conformations of retinals are calculated randomly by GOLD [21]. However, double bonds are treated as rigid even with full flexibility allowed for the retinals during the docking process. GOLD does therefore generally not alter stereochemistry. The torsion angles selected by GOLD to determine ‘favorable’ versus ‘unfavorable’ distributions are taken from the Cambridge Structural Database (http://www.ccdc.cam.ac.uk/products/csd/) that records biblio-graphic, chemical and crystallographic information for small organic molecules. As a result, even with full flexible single bonds, a set of stable conformations of the retinals is automatically selected as input structures for the docking process. During the docking procedure an internal energy term is calculated by GOLD for each docked ligand pose, which is a component of the scoring function used to rank the pose. This internal energy calculation estimates steric clashes within a conformation, as well as torsional energy. In summary, there is a bias towards stable retinal conformations during the docking.

### Examination of docking

GOLD is based on a genetic algorithm to explore the full range of ligand conformational flexibility with partial flexibility of the receptor [21]. We have used the *GoldScore* (search efficiency: 100%, no additional constraints) as the scoring function, where the simulated annealing parameters, van der Waals and hydrogen bonding, allow weak hydrogen bonds or van der Waals contacts to occur at the beginning of a genetic algorithm run, in the expectation that they will evolve to better solutions. The function (and final ranking of the ligand poses) is based of four components, namely **(i)** protein-ligand hydrogen bond energy, **(ii)** protein-ligand van der Waals energy, **(iii)** ligand internal van der Waals energy and **(iv)** ligand torsional strain energy. Following the completion of all docking runs, the results were compared by a hierarchical cluster analysis implemented in GOLD (Figures 5–7). The RMSD (root mean square deviation) algorithm thereby applied takes account of symmetry effects, using a graph isomorphism algorithm [21]. The final complex structures were always selected from the top ten lists ranking the energetically most plausible docking modes of each run (according to the above described scoring function). The docking mode with the highest score was regularly chosen, except for those instances, where the ligand was not docked in the correct longitudinal orientation, e.g. with retinal being docked the other way around in the binding pocket. In that case, the docking mode with the highest score from the next cluster was taken.
